# Performance Deterioration of Asphalt Mixture under Chloride Salt Erosion

**DOI:** 10.3390/ma14123339

**Published:** 2021-06-17

**Authors:** Fuyu Wang, Xingyuan Qin, Weichen Pang, Wensheng Wang

**Affiliations:** 1 College of Transportation, Jilin University, Changchun 130025, China; wfy@jlu.edu.cn (F.W.); xingyuant20@mails.jlu.edu.cn (X.Q.); pangwc19@mails.jlu.edu.cn (W.P.); 2 College of Construction Engineering, Jilin University, Changchun 130026, China

**Keywords:** asphalt mixture, salt erosion, freeze-thaw cycles, performance evaluation, viscoelastic behavior

## Abstract

In order to ensure smooth traffic and driving safety, deicing salt or snow melting agents are usually adopted to solve the problem of traffic jams and prevent pavement surfaces from freezing. The objective of this present study is to investigate the performance deterioration evaluation of asphalt mixture under the chloride salt erosion environment. Five chloride salt solution concentrations were designed and the uniaxial static compression creep test, low-temperature IDT test, freeze-thaw splitting test, and freeze-thaw cycle test were carried out for asphalt mixtures (AC-16) soaked in chloride salt solution. Results showed that with the increase in chloride salt solution concentration, the high-temperature stability, low-temperature crack resistance, and water stability of the asphalt mixture decreases. Moreover, the high-temperature stability, low-temperature crack resistance, and water stability of the asphalt mixture show a decreasing trend under different chloride salt solution concentrations following the negative cubic polynomial function. Based on the viscoelastic analysis, chloride salt solution could reduce the ability of the asphalt mixture to resist instantaneous elastic deformation and permanent deformation, and this influence will become more obvious with the increase in chloride salt solution concentration. In addition, the salt freeze-thaw cycle test indicated that in the early stage of freeze-thaw cycles, the splitting tensile strength of the asphalt mixture decreases rapidly, then tends to be flat, and then decreases rapidly. This study explores the performance damage law of asphalt mixture under salt corrosion, and the analysis results of this study could provide some references for the chloride salt dosage in the snow melting project while spreading deicing salt.

## 1. Introduction

In northern China, the temperature is very low in winter (the average temperature is about −15 °C); the duration of freezing is long, then continuous snowfall or heavy snow easily leads to traffic jams on expressways and urban roads [1,2,3,4,5]. The decrease in the anti-sliding performance of asphalt pavement may even lead to serious traffic accidents [6,7,8]. In order to ensure smooth traffic and driving safety, many road projects adopt the method of spreading deicing salt, a snow melting agent with the function of quick deicing and snow melting, to solve the problem of traffic jams and prevent pavement surfaces from freezing [9]. However, the deicing salt solution would gradually penetrate the interior of the asphalt mixture in the different work environments, and the migration of chloride ions follows Fick’s second law, resulting in a series of physical and chemical reactions (such as the salting-out effect and condensation reaction), leading to asphalt softening, asphalt film spalling, aggregate looseness, and other distress [10,11,12,13].

The main working principle of spreading the deicing salt snow melting agent is based on the freezing point reduction theory [14,15]. The so-called freezing point refers to the temperature when a substance reaches the state of solid-liquid coexistence. When chloride salts are added into water, the surface of water is occupied by chloride salt ions, resulting in relatively small vapor pressure of water compared with ice. Therefore, it is necessary to lower the temperature to make the vapor pressure of ice equal to the vapor pressure of water, resulting in a decrease in the freezing point. Asphalt mixture is a multi-phase mixture system composed of coarse and fine aggregates, fillers, and asphalt binders [8]. Its strength formation comes from two aspects: on the one hand, it is the embedment and extrusion between aggregates; on the other hand, it is the bonding effect of asphalt binders [15,16,17].

At present, many scholars have carried out extensive research on the performance damage of asphalt mixture under salt solution. Zhou et al. investigated the failure influences of salt erosion on the interfacial effect between asphalt and aggregate at the conditions of high temperature and hygrothermal environments [18]. Xu et al. investigated the transport property of chloride salt in asphalt mixtures under different working conditions based on Fick’s second law [19]. Ran et al. used ABAQUS software to simulate the 45° shear test for the contact state between the base layer and asphalt layer under the effects of different salt solution concentrations and temperatures [20]. Xiong et al. carried out the durability evaluation of asphalt mixture in the salt corrosion condition under the effect of dynamic water pressure via a splitting test [21]. Zhang et al. studied the influences of salt erosion conditions on the asphalt binder by using a four fractions test and an atomic force microscopy, and they found that the main reason for the performance degradation of asphalt binder is that the chemical composition of asphalt binder changed under the action of salt erosion, resulting in the phenomenon of salt aging. At the same time, the freeze-thaw cycle test of salt solution was simulated [22]. Xiong et al. utilized CT technology to study the void characteristics of asphalt mixture under the action of salt erosion by using a three-dimensional reconstruction model. They found that there is a linear relationship between the splitting strength of asphalt mixture in the salt erosion condition and its void characteristics. The crystallization pressure caused by salt will accelerate the development of internal pores of asphalt mixture, resulting in a decrease in its mechanical properties [14]. Wang et al. investigated the pavement performances of asphalt mixture containing salt-storage aggregates with the function of snow melting [9]. Liu et al. evaluated the antifreeze and moisture susceptibility of asphalt mixture containing salt-based filler and its thermal analysis and scanning electron microscope [23].

In the seasonal frozen area of northern China, asphalt pavement performance failure occurs under the combined action of repeated freeze-thaw and chloride salt erosion, which leads to the further development of water damage of asphalt pavement [10,12,16]. With respect to the influence of freeze-thaw cycle in salt erosion conditions on the performance of asphalt mixture, Cui et al. investigated the viscoelastic properties of asphalt mortar in the salt and freezing condition and used the Burgers model while considering the damage factor to analyze the viscoelastic properties of asphalt mortar. Meanwhile, the microstructure of asphalt binder in the salt and freezing condition was studied by scanning electron microscopy [24]. Guo et al. explored the interface bonding property between asphalt and aggregate subjected freeze-thaw cycles and salt solution erosion through pull-off tensile tests considering different test temperatures [15]. Zhang et al. studied the influences of chlorine salt and freeze-thaw cycles on asphalt mastics from the perspective of spectroscopic and characteristics by using Fourier-transform infrared spectroscopy, gel permeation chromatography, and atomic force microscopy techniques [25]. Amini et al. investigated the influences of moisture and chloride salt on the performance degradation of asphalt mixture subjected to freeze-thaw cycles according to the Marshall stability and mass loss [26]. Feng et al. discussed the effects of salt on asphalt binder based on the conventional performances, and further studied the influences of salt and freeze-thaw cycles on the mechanical and volumetrics of asphalt mixes. They found that the freeze-thaw cycle effect is the main factor influencing asphalt mixture damage, and chloride salt erosion would accelerate the damage of the asphalt mixture [27]. However, the performance deterioration of asphalt mixture varying in the wide range of chloride salt concentration was not considered.

In this study, asphalt mixture was firstly prepared by using the Marshall test method, and then various chloride salt solution concentrations were designed to simulate the chloride salt environment of an asphalt pavement with deicing salt in winter. Subsequently, the uniaxial static compression creep test (for high-temperature stability), low-temperature IDT test (for crack resistance), freeze-thaw splitting test (for water stability), and freeze-thaw cycle test (for freeze-thaw resistance) were carried out on asphalt mixtures which were soaked in chloride salt solution, to evaluate the performance deterioration of the asphalt mixture. The analysis and results of this study could provide some references for the chloride salt dosage in the snow melting project.

## 2. Materials

### 2.1. Asphalt Binder

The asphalt binder used in this study was an unmodified asphalt binder (Zhonghai AH-70, Zhonghai Bitumen Co., Ltd., Binzhou, China). According to the Chinese specification requirements of “Standard test methods of bitumen and bituminous mixtures for highway engineering” (JTG E20-2011) [28] and “Technical specifications for construction of highway asphalt pavements” (JTG F40-2004) [29], the AH-70 asphalt binder has been tested in the laboratory, and its basic performance parameters are shown in Table 1. The test results show that the AH-70 asphalt binder meets the specification requirements.

### 2.2. Aggregates

The limestone aggregates used in this study were sieved and tested according to the Chinese specification of “Test methods of aggregate for highway engineering” (JTG E42-2005) [26,30]. The test results are shown in Table 2, Table 3 and Table 4. The test results show that the coarse aggregate, fine aggregate, and mineral powder meet the specification requirements.

### 2.3. Snow-Melting Salt

According to the Chinese specification of “Salt of ice and snow melting for road” (GB/T 23851-2009) [31], the ability of snow melting salt should reach 90% of that of sodium chloride, that is to say, sodium chloride can be used as a standard to evaluate the snow melting ability of snow-melting salt. Therefore, this study used sodium chloride snow melting salt as the main research object to study the influence of chloride salt concentration on the performance of the asphalt mixture. After testing, the dissolution rate of chloride salt (snow melting salt) used in this study is 6.955 g/min at standard atmospheric pressure and stirring rate of 100 r/min, which meets the specification requirements.

### 2.4. Asphalt Mixture Preparation

**Figure 1:**
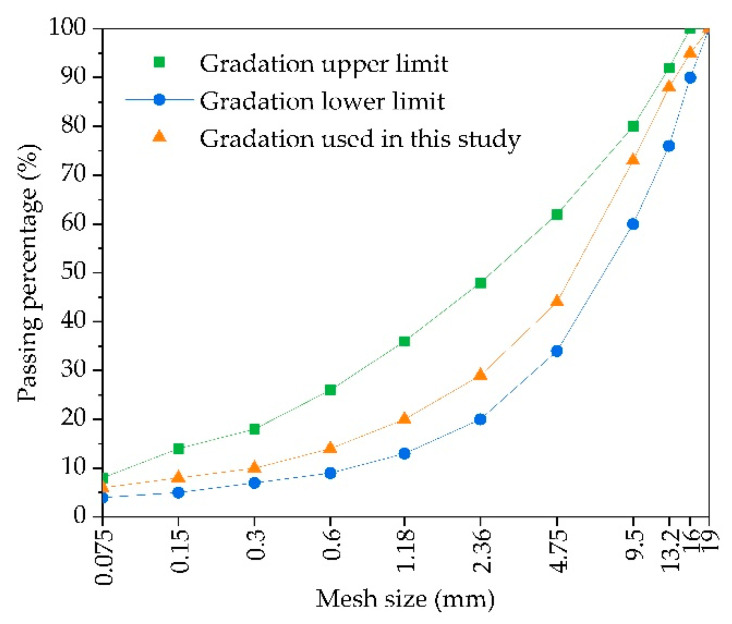
The mineral aggregate gradation curve of AC-16.

The main purpose of asphalt mixture design is to determine the mineral aggregate gradation and the optimal asphalt content in asphalt mixture. According to “Technical specifications for construction of highway asphalt pavements” (JTG F40-2004) [29], combined with the engineering experience, the mineral aggregate gradation of asphalt mixture (AC-16) used in this study was determined as the median gradation, and the mineral aggregate gradation is shown in Figure 1.

The Marshall design method is a mature method to determine the optimum asphalt content [32,33,34]. The comprehensive performance of asphalt mixture is usually the best under the optimal asphalt content. According to the determined mineral aggregate gradation composition, the asphalt content was selected in the range of 4.6~5.8%, and the Marshall specimens were made with four different asphalt contents at an interval of 0.4%. According to the compaction method (T0702) of “Standard test methods of bitumen and bituminous mixtures for highway engineering” (JTG E20-2011) [28], the asphalt mixture AC-16 specimens were compacted 75 times on both sides, then the optimal asphalt content could be determined.

### 2.5. Optimal Asphalt Content

It is known that asphalt content has a great influence on the road performances of asphalt mixture [32]. At larger asphalt content, the high-temperature deformation resistance of asphalt mixture would decrease, and asphalt pavement is prone to rutting, bleeding, and other distress. While at smaller asphalt content, the cohesive force between asphalt and aggregates will also be reduced, and asphalt pavement is prone to aging, water damage, etc. Therefore, the optimal asphalt content is generally firstly determined. In this study, the standard Marshall test method has been used to determine the optimal asphalt content of asphalt mixture.

**Figure 2:**
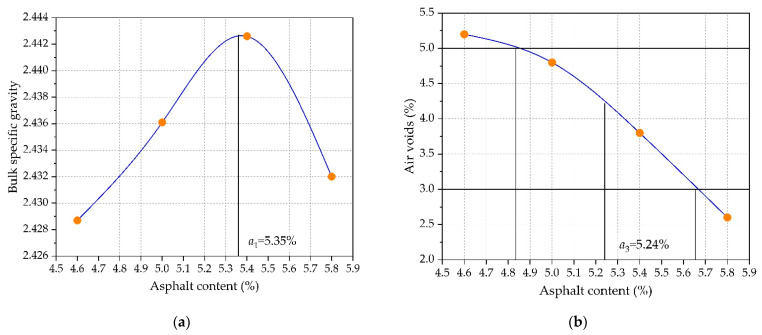

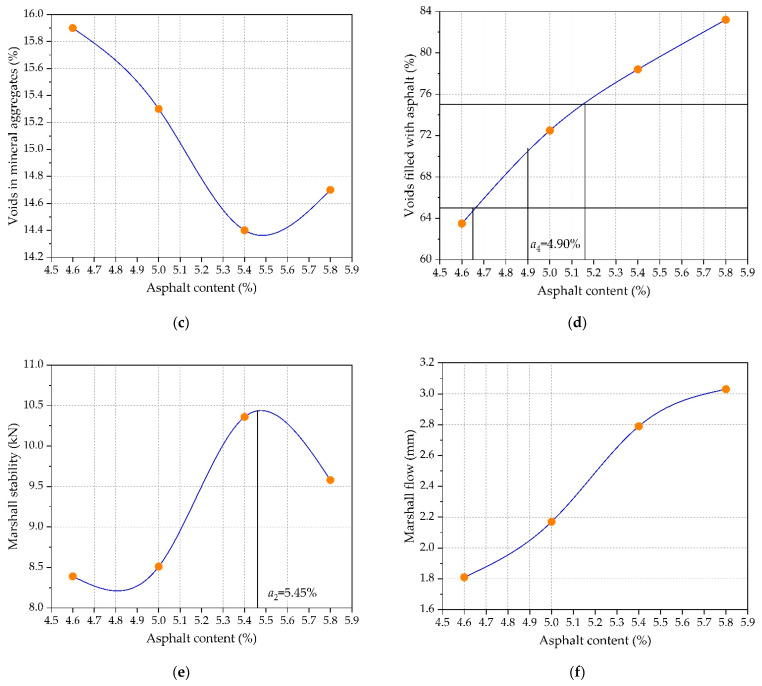
Marshall mix design results of AC-13: (**a**) bulk specific gravity; (**b**) air voids; (**c**) voids in mineral aggregates; (**d**) voids filled with asphalt; (**e**) Marshall stability; and (**f**) Marshall flow.

According to the Chinese specification requirements of “Standard test methods of bitumen and bituminous mixtures for highway engineering” (JTG E20-2011) [28], the bulk specific gravity, air voids, voids in mineral aggregates, voids filled with asphalt, Marshall stability, and flow value could be measured for the prepared standard Marshall specimens, as shown in Figure 2. Then, the optimal asphalt content of AC-16 would be determined at the maximum bulk specific gravity and Marshall stability, median air voids, and voids filled with asphalt. According to “Technical specifications for construction of highway asphalt pavements” (JTG F40-2004) [29], OAC_1_ = (a_1_ + a_2_ + a_3_ + a_4_)/4 = 5.23%, OAC_1_ = (OAC_min_ + OAC_max_)/2 = 5.10%, and OAC = (OAC_1_ + OAC_2_)/2 = 5.16%. Therefore, the optimal asphalt content in this study was 5.16% based on the Marshall design results.

## 3. Methods

### 3.1. High-Temperature Stability

The high-temperature stability of asphalt mixture refers to the characteristic of resisting the repeated action of vehicle load without significant deformation under the condition of high temperature. Due to the lack of high-temperature stability, there are some asphalt pavement distresses including rutting, side shift, disintegration, bleeding, and so on, of which rutting is the most common distress [35]. The most widely used and mature methods for high-temperature stability of asphalt mixture are rutting test and uniaxial static creep test, which have been described in previous studies [13,36].

In this paper, the NU-14 type pneumatic asphalt multifunctional testing machine, produced by British Cooper company, UK, was used to evaluate the influence of different concentrations of chloride snow melting salt on the high-temperature stability of asphalt mixture by uniaxial creep test. The specific test steps are as follows:

- Step 1: specimen pretreatment
  Asphalt mixture specimens were put into water and sodium chloride solution (6%, 12%, 18%, and 24%), in which three specimen replicates should be immersed by liquid to make the specimens fully saturated. After 7 days of full submersion, the asphalt mixture specimens were taken out and placed in the dry place for 1 day;
- Step 2: temperature control of specimen
  The uniaxial creep test temperature was set as 50 °C, and pretreated asphalt mixture specimens were put into a temperature control chamber for at least 4 h;
- Step 3: uniaxial creep test
  After the asphalt mixture specimen was coupled with the upper and lower pressure plates, the LVDT sensors were connected and the test parameters were set, that is, the axial stress was 10% (0.2 MPa) of the failure load, and the loading time was 2700 s. In order to eliminate the contact voids, the preload of 300 s with the stress of 10 kPa (5% of the test loading stress) was applied before the test.

### 3.2. Low-Temperature Crack Resistance

The low-temperature crack resistance of asphalt mixture usually refers to its ability to resist shrinkage deformation [37]. The development of cracks will damage the smoothness and reduce the bearing capacity of asphalt pavement, but also easily lead to other issues. For example, water will penetrate the pavement structure layer along the cracks in areas with more rain and snow, and then the subgrade and pavement structure will lose the bearing capacity due to a long-term freeze-thaw cycle [16,17].

As the indirect tensile (IDT) strength test is relatively simple, the low-temperature IDT test was used to study the influence of different concentrations of chloride snow melting salt on the low-temperature crack resistance of asphalt mixture. The specific test steps are as follows:

- Step 1: specimen pretreatment
  The specimen pretreatment of low-temperature IDT test is similar to that of the above high-temperature uniaxial creep test;
- Step 2: temperature control of specimen
  The pretreated asphalt mixture specimens were put into a constant temperature chamber of −10 °C for at least 6 h;
- Step 3: IDT test
  Low-temperature IDT test of pretreated Marshall asphalt mixture specimens was carried out by using a pavement material strength tester, and the time from taking specimens out the chamber to the end of test should not exceed 45 s.

### 3.3. Water Stability

Generally, the ability of asphalt mixture to resist water erosion is known as the water stability of asphalt mixture [5,38]. The asphalt pavement damage caused by the lack of water stability has become one of the main distresses for asphalt pavement in China [27]. In the northern region, chloride snow melting salt is often used in winter to maintain smooth traffic, so asphalt pavement is often under the combined action of water, temperature, and chloride snow melting salt [39]. In this study, the freeze-thaw splitting test was used to study the relationship between the concentration of chloride snow melting salt and the water stability of the asphalt mixture. The specific test steps are as follows:

- Step 1: specimen pretreatment
  The solution involved in the freeze-thaw test would be replaced by chloride solution of different concentrations, such as saturated water and water bath, etc.;
- Step 2: freeze-thaw splitting test
  The saturated asphalt mixture specimens were put into plastic bags with 10 mL chloride solution, and then stored in the refrigerator at the required temperature for 16 h. After that, the asphalt mixture specimens were put into the water bath for 24 h. Four specimen replicates should be prepared for each group of sodium chloride solutions. After the freeze-thaw cycle, the splitting test was carried out, as described above.

## 4. Results and Discussion

### 4.1. High-Temperature Stability

#### 4.1.1. Uniaxial Compressive Creep Test Results of AC-16

**Figure 3:**
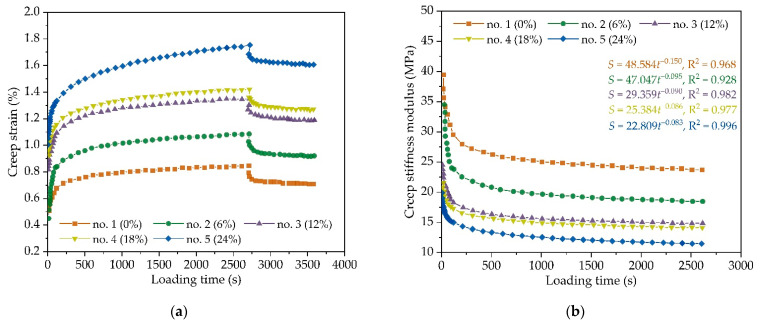
The uniaxial compressive creep test results of AC-16 under different chloride salt concentrations: (**a**) creep strain; and (**b**) creep stiffness modulus.

According to the uniaxial compressive creep test at 50 °C, the creep deformation curves of AC-16 under different chloride salt concentrations are shown in Figure 3a. Figure 3a demonstrates that the strain curves of the uniaxial static compressive creep test of AC-16 can be obviously divided into three stages.

- Stage I: preloading stage
  In stage I, the slopes of creep strain curves are larger, and the loading time is shorter. The creep curves are almost perpendicular to the abscissa axis and an approximate straight line. This is as asphalt mixture, as a kind of viscoelastic material, is subjected to vertical load, showing instantaneous elastic performance in a short time, and has a high deformation rate.
- Stage II: constant loading stage
  In stage II, the creep strain curves are relatively gentle, and the slope changes little. Meanwhile, the creep strain of the asphalt mixture specimens increases gradually with the increase in loading time, which is also called the stable stage.
- Stage III: unloading stage
  The deformation of the asphalt mixture specimens has obvious recovery. This is due to the viscoelastic characteristics of asphalt mixture, when the constant loading disappears, the instantaneous elastic deformation of the asphalt mixture will gradually recover with time, and the viscous flow deformation will become a permanent deformation.

In addition, comparison among different groups shows that the residual deformation of asphalt mixtures after chloride salt erosion is significantly greater than that of the control group. Meanwhile, the permanent deformation of asphalt mixture increases with the increase in chloride solution concentration, implying that the high-temperature performance of asphalt mixture decreases. This could be attributed to the emulsification effect between chloride salt and asphalt when the chloride salt solution penetrates the asphalt mixture through the air voids. The chloride salt erosion would reduce the adhesive properties between asphalt and aggregate to a certain extent, leading to the decrease in the shear strength of the asphalt mixture. With the increase in chloride solution concentration, the emulsification of chloride salt and asphalt will be further intensified, and the high-temperature performance of the asphalt mixture will be reduced.

Figure 3b shows the creep stiffness modulus-loading time curves of asphalt mixture under different chloride solution concentrations. The creep stiffness modulus of asphalt mixture specimens appears to decrease very quickly in the preloading stage, and then the curves of creep stiffness modulus gradually flatten until the unloading stage, which indicates the viscoelastic characteristics of the asphalt mixture. The comparison among different groups demonstrates that the creep stiffness modulus of asphalt mixture affected by chloride salt erosion is significantly smaller than that of control group. Due to the emulsification effect between chloride salt and asphalt, the creep stiffness modulus of asphalt mixture decreases with the increase in chloride solution concentration. According to the statistical analysis, the curves of creep stiffness modulus of asphalt mixture with loading time can be fitted by a power function. From the fitting power equations in Figure 3b, the absolute value of the fitting power index decreases with the chloride solution concentration, which indicates that chloride salt erosion will reduce the reduction rate of creep stiffness modulus.

#### 4.1.2. Creep Model Analysis Based on Burgers Model and Modified Burgers Model

**Figure 4:**
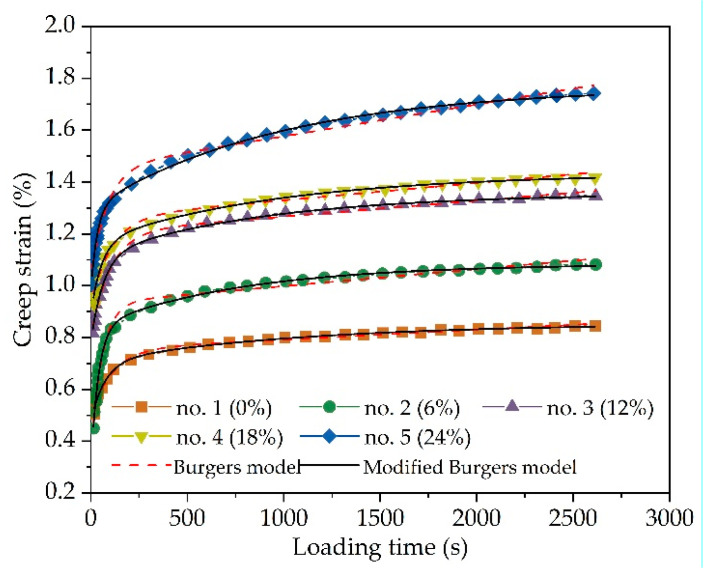
Comparison of Burgers model and modified Burgers model of AC-16 under different chloride salt concentrations.

Asphalt mixture is a typical viscoelastic material, and the constitutive equation of the viscoelastic model is used to study the creep behavior of asphalt mixture. Two commonly used viscoelastic models, i.e., the Burgers model and modified Burgers model, are adopted to fit the above-measured creep deformation curves. The Burgers model and modified Burgers model have been described in detail in previous studies [34,40,41], and are given in Equations (1) and (2).

(1) $$\mathrm{Burgers}\;\mathrm{model}:\;\varepsilon (t)=\sigma_{0}\left[\frac{1}{E_{1}}+\frac{t}{\eta_{1}}+\frac{1}{E_{2}}\left(1-e^{-E_{2}t/\eta_{2}}\right)\right]$$

(2) $$\mathrm{Modified}\;\mathrm{Burgers}\;\mathrm{model}:\;\varepsilon (t)=\sigma_{0}\left[\frac{1}{E_{1}}+\frac{\left(1-e^{-Bt}\right)}{AB}+\frac{1}{E_{2}}\left(1-e^{-E_{2}t/\eta_{2}}\right)\right]$$

where *E*_1_, *E*_2_, *η*_1_, and *η*_2_ are viscoelastic parameters, and *A* and *B* are fitting constants. Figure 4 plots the fitting Burgers model and modified Burgers model of AC-16 under different chloride salt concentrations at the loading stage. The fitting Burgers model and modified Burgers model possess a high accuracy with R^2^ above 0.99. This indicates that both the Burgers model and modified Burgers are close to actual creep strain curves of asphalt mixture.

As a viscoelastic material, asphalt mixture has creep deformation under constant loading. *E*_1_ represents the instantaneous elastic deformation modulus in the Maxwell mechanical model, which will recover immediately after the external load disappears. The larger the value of *E*_1_, the better the ability of the asphalt mixture to resist instantaneous elastic deformation. *E*_2_ and *η*_2_ represent the elastic modulus and viscosity coefficient under long-term load in the Kelvin mechanical model, which will recover slowly after the external load disappears. *η*_1_ represents the viscosity coefficient of the Maxwell mechanical model in the process of loading, which is irrecoverable. The greater the value of *η*_1_, the better the ability of the asphalt mixture to resist permanent deformation. In the modified Burgers model, the product of fitting parameters *A* and *B* could be used to characterize the ability of asphalt mixture to resist permanent deformation.

**Figure 5:**
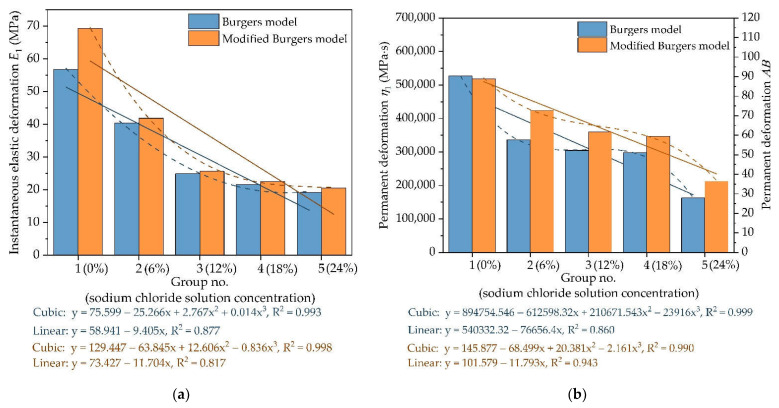
The variation of viscoelastic parameters with different chloride salt concentrations: (**a**) instantaneous elastic modulus; and (**b**) viscous resistance.

The variation of viscoelastic parameters of AC-16 under different chloride salt concentrations is plotted in Figure 5. Figure 5 shows that the instantaneous elastic modulus (*E*_1_) in the Burgers model and modified Burgers model would decrease with chloride salt concentrations. Through the comparative analysis of different fitting models, the cubic polynomial function is more suitable to fit the variation of instantaneous elastic modulus of AC-16 under different chloride salt concentrations, in which R^2^ of a cubic polynomial function is higher than that of a linear function. As shown in Figure 5b regarding the variation of viscous resistance, the permanent deformation (*η*_1_) in the Burgers model has a similar trend with *AB* in the modified Burgers model. Compared with a linear function, the permanent deformation *η*_1_ and *AB* decreases following the negative cubic polynomial function. Therefore, chloride salt solution could reduce the ability of asphalt mixture to resist instantaneous elastic deformation and permanent deformation, and this influence will become more obvious with the increase in chloride salt solution concentration.

### 4.2. Low-Temperature Crack Resistance

**Figure 6:**
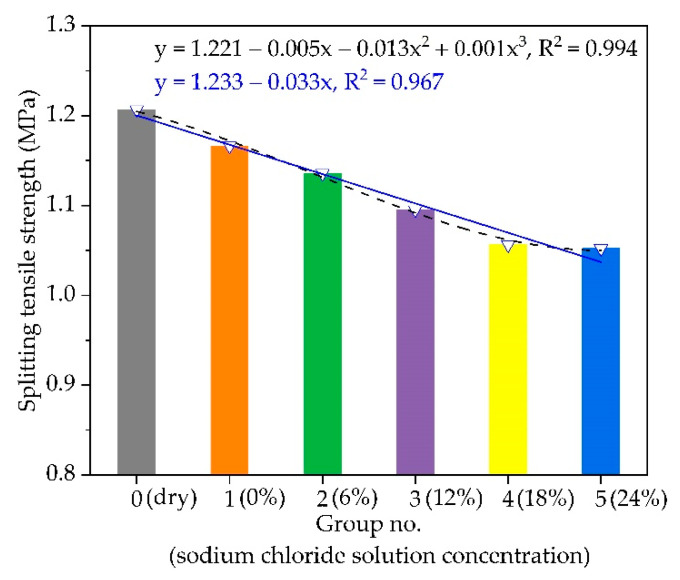
The variation of low-temperature IDT strength with different chloride salt concentrations.

The low-temperature IDT tensile strength of AC-16 under different chloride salt concentrations is plotted in Figure 6. This figure highlights that the low-temperature crack resistance of asphalt mixture specimens soaked in water and chloride salt solution is reduced, and the influence of chloride salt solution on the asphalt mixture specimens is more serious than that of water. After soaked in a chloride salt solution of 0%, 6%, 12%, 18%, or 24%, the IDT tensile strength of asphalt mixture specimens are reduced by 3.4%, 5.9%, 9.2%, 14.3%, and 23.2%, respectively, compared to the control group (i.e., dry AC-16 specimen). In Figure 6, the IDT tensile strength of the asphalt mixture decreases with the increase in chloride solution concentration. When the chloride solution concentration is greater than 12%, the slope of the curve increases, indicating that the effect of chloride solution on the IDT tensile strength of asphalt mixture becomes more obvious when the chloride solution concentration is greater than 12%. The low-temperature IDT strength of AC-16 decreases under different chloride salt concentrations following the negative cubic polynomial function. The chloride salt solution enters the asphalt mixture via the air voids, erodes the interface between asphalt and aggregate, reduces their adhesion, and weakens the interaction between asphalt and aggregate. This is due to the salting-out effect and condensation reaction between asphalt and chloride salt [16,42]. When the chloride salt solution concentration increases, the concentration of external ions increases, and chloride ions are more likely to enter the asphalt mixture and cause damage under capillary action due to the higher solution concentration.

### 4.3. Water Stability

**Figure 7:**
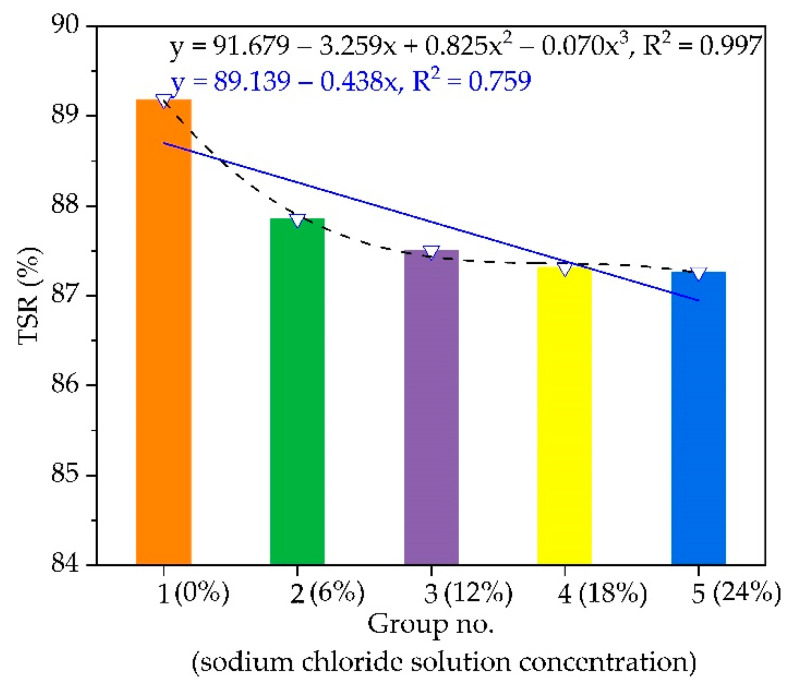
The variation of TSR with different chloride salt concentrations.

The freeze-thaw splitting tensile strength ratio (TSR) of AC-16 under different chloride salt concentrations is plotted in Figure 7. The water stability of asphalt mixture specimens soaked in water (0%) and chloride salt solution (6%, 12%, 18%, and 24%) is reduced with the increase in sodium chloride solution concentration. The TSR results of the asphalt mixture specimens soaked in chloride salt solution of 6%, 12%, 18%, and 24%, are reduced by 1.4%, 1.8%, 2.0%, and 2.2%, respectively, compared to the asphalt mixture soaked in water (0%). When the chloride solution concentration is greater than 6%, the slope of the curve increases, meaning that the effect of chloride solution on the water stability of the asphalt mixture becomes more obvious. Meanwhile, the water stability of AC-16 decreases under different chloride salt concentrations following the negative cubic polynomial function. The variation trend of TSR with chloride salt concentration changes, first quickly, then slowly. This may be due to the chloride salt solution entering the asphalt mixture from the air voids, eroding the interface between asphalt and aggregate.

### 4.4. Freeze-Thaw Resistance

**Figure 8:**
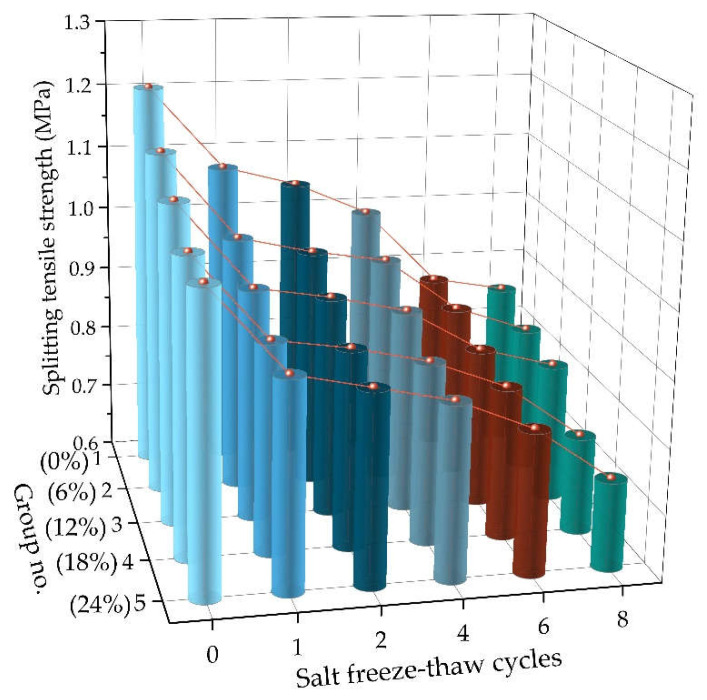
The variation of splitting tensile strength with freeze-thaw cycles under different chloride salt concentrations.

Considering the frequent rain and snow in northeast China in winter, asphalt pavement will experience more than one freeze-thaw cycle after spraying snow melting salt. In order to simulate the long-term effect of snow melting chloride salt on asphalt pavement, asphalt mixtures were conducted through 1, 2, 4, 6, and 8 freeze-thaw cycles to study the mechanical properties. Figure 8 presents the variation of splitting tensile strength with freeze-thaw cycles for AC-16 specimens under different chloride salt concentrations.

As seen in Figure 8, the splitting tensile strength of the asphalt mixtures decreases with the increase in freeze-thaw cycles, following a cubic polynomial decrease for the variation trend. In the early stage of freeze-thaw cycles, the splitting tensile strength of the asphalt mixture decreases rapidly, then tends to be flat, and finally decreases quickly again. Additionally, the splitting tensile strength of asphalt mixture specimens soaked in water is higher than that soaked in chloride salt solution, and the splitting tensile strength of asphalt mixture decreases with the increase in chloride solution concentration, showing a similar variation trend in the low-temperature crack resistance. Indeed, when asphalt mixture specimens are below 0 °C, water entering asphalt mixture will freeze into an ice state, which causes an increased volume due to the frost heaving, and then some fine cracks would occur in the asphalt mixture structure. When the temperature of asphalt mixture specimens is above 0 °C, the ice inside the asphalt mixture structure will melt into water, which penetrates along the air voids or cracks on the surface of the asphalt mixture specimens and the internal capillary, causing deep damage to the asphalt mixture. With the increase in freeze-thaw cycles, the freeze-thaw effect occurs alternately, damaging the internal structure of the asphalt mixture and reducing its splitting tensile strength. On the other hand, chloride salt solution reduces the adhesion between asphalt and aggregates, resulting in a significant weakening of the interaction and a decrease in the mechanical strength of the asphalt mixture. With the promotion of freeze-thaw cycles, the penetration speed of chloride salt solution to the interface between asphalt and aggregate would accelerate and cause greater damage to the asphalt mixture.

## 5. Conclusions

In this study, the asphalt mixture specimens soaked with different snow melting chloride salt concentrations were taken as the research object. The uniaxial static compression creep test, low-temperature IDT test, freeze-thaw splitting test, and freeze-thaw cycle test were carried out. The viscoelastic theory was used to analyze the creep test of asphalt mixture. The conclusions are as follows:

(1) With the increase in chloride salt solution concentration, the high-temperature stability, low-temperature crack resistance, and water stability of asphalt mixture decrease. Moreover, the high-temperature stability, low-temperature crack resistance, and water stability of the asphalt mixture show a decreasing trend under different chloride salt solution concentrations following a negative cubic polynomial function;
(2) The instantaneous elastic modulus (*E*_1_) and the permanent deformation (*η*_1_ and *AB*) decrease following a negative cubic polynomial function. In addition, chloride salt solution could reduce the ability of the asphalt mixture to resist instantaneous elastic deformation and permanent deformation, and this influence will become more obvious with an increase in chloride salt solution concentration;
(3) In this study, the salt freeze-thaw cycle test was used to simulate the long-term effect of snow melting chloride salt on asphalt pavement. The IDT strength of asphalt mixtures decreases with the increase in salt freeze-thaw cycles, presenting a negative cubic polynomial decreasing trend. In the early stage of freeze-thaw cycles, the splitting tensile strength of asphalt mixture decreases rapidly, then tends to be flat, and finally decreases quickly again.

In the follow-up work, we will carry out micro tests to explore the mechanism of the effect of snow melting chloride salt on the performance of asphalt mixture, and verify the snow melting chloride salt amount suggested in this study, combined with engineering practice. In addition, the relationships between the different parameters would be discussed.

## Figures and Tables

**Table 1:** Basic performance parameters of AH-70 asphalt binder.

Test Items	Units	Index	Requirements
Penetration @ 25 °C	0.1 mm	75	60~80
Penetration index (PI)	-	−1.77	−1.8~+1.0
Ductility @ 10 °C	cm	25.4	≥20
Ductility @ 15 °C	cm	>100	≥100
Softening point (R&B)	°C	46.7	≥43
Brookfield viscosity @ 135 °C	Pa∙s	0.527	-
Brookfield viscosity @ 60 °C	Pa∙s	0.793	-

**Table 2:** Performance parameters of coarse aggregate.

Particle Size (mm)	16	13.2	9.5	4.75
Bulk density (g/cm^3^)	2.851	2.856	2.811	2.768
Saturated surface-dry density (g/cm^3^)	2.875	2.886	2.833	2.808
Apparent density (g/cm^3^)	2.915	2.944	2.905	2.876
Water absorption (%)	0.96	1.02	1.28	1.48
Weared stone value (%)	Average: 8.41			
Flat and elongated particle content (%)	Average: 7.66			
Crushed stone value (%)	Average: 19.81			

**Table 3:** Performance parameters of fine aggregate.

Particle Size (mm)	2.36	0~2.36
Bulk density (g/cm^3^)	2.727	2.215
Saturated surface-dry density (g/cm^3^)	2.769	2.751
Apparent density (g/cm^3^)	2.842	2.827
Water absorption (%)	1.49	1.55

**Table 4:** Performance parameters of mineral powder.

Test Items	Index	Requirements
Apparent specific density	2.763	≥2.450
Hydrophilic coefficient	0.87	<1
Water absorption (%)	0.91	≤1
Passing percentage <0.6 mm (%)	100.0	>98.6
Passing percentage <0.15 mm (%)	92.4	>78.5
Passing percentage <0.075 mm (%)	75.5	>62.2

## Data Availability

The data presented in this study are available on request from the corresponding author.
